# Antibiotic-Loaded Gold Nanoparticles: A Nano-Arsenal against ESBL Producer-Resistant Pathogens

**DOI:** 10.3390/pharmaceutics15020430

**Published:** 2023-01-28

**Authors:** Syed Mohd Danish Rizvi, Amr Selim Abu Lila, Afrasim Moin, Talib Hussain, Mohammad Amjad Kamal, Hana Sonbol, El-Sayed Khafagy

**Affiliations:** 1 Department of Pharmaceutics, College of Pharmacy, University of Ha’il, Ha’il 81442, Saudi Arabia; 2 Molecular Diagnostic & Personalized Therapeutic Unit, University of Ha’il, Ha’il 81442, Saudi Arabia; 3 Department of Pharmacology and Toxicology, College of Pharmacy, University of Ha’il, Ha’il 81442, Saudi Arabia; 4 Institutes for Systems Genetics, Frontiers Science Center for Disease-Related Molecular Network, West China Hospital, Sichuan University, Chengdu 610065, China; 5 King Fahd Medical Research Center, King Abdulaziz University, Jeddah 21589, Saudi Arabia; 6 Department of Pharmacy, Faculty of Allied Health Sciences, Daffodil International University, Dhaka 1207, Bangladesh; 7 Enzymoics, Novel Global Community Educational Foundation, 7 Peterlee Place, Hebersham, NSW 2770, Australia; 8 Department of Biology, College of Science, Princess Nourah bint Abdulrahman University, Riyadh 11671, Saudi Arabia; 9 Department of Pharmaceutics, College of Pharmacy, Prince Sattam Bin Abdulaziz University, Al-kharj 11942, Saudi Arabia; 10 Department of Pharmaceutics and Industrial Pharmacy, Faculty of Pharmacy, Suez Canal University, Ismailia 41522, Egypt

**Keywords:** antibiotic resistance, bacterial pathogens, ESBLs, gold nanoparticles, nano-therapeutics

## Abstract

The advent of new antibiotics has helped clinicians to control severe bacterial infections. Despite this, inappropriate and redundant use of antibiotics, inadequate diagnosis, and smart resistant mechanisms developed by pathogens sometimes lead to the failure of treatment strategies. The genotypic analysis of clinical samples revealed that the rapid spread of extended-spectrum β-lactamases (ESBLs) genes is one of the most common approaches acquired by bacterial pathogens to become resistant. The scenario compelled the researchers to prioritize the design and development of novel and effective therapeutic options. Nanotechnology has emerged as a plausible groundbreaking tool against resistant infectious pathogens. Numerous reports suggested that inorganic nanomaterials, specifically gold nanoparticles (AuNPs), have converted unresponsive antibiotics into potent ones against multi-drug resistant pathogenic strains. Interestingly, after almost two decades of exhaustive preclinical evaluations, AuNPs are gradually progressively moving ahead toward clinical evaluations. However, the mechanistic aspects of the antibacterial action of AuNPs remain an unsolved puzzle for the scientific fraternity. Thus, the review covers state-of-the-art investigations pertaining to the efficacy of AuNPs as a tool to overcome ESBLs acquired resistance, their applicability and toxicity perspectives, and the revelation of the most appropriate proposed mechanism of action. Conclusively, the trend suggested that antibiotic-loaded AuNPs could be developed into a promising interventional strategy to limit and overcome the concerns of antibiotic-resistance.

## 1. Introduction

Bacterial pathogens produce a class of enzymes known as extended-spectrum β-lactamases (ESBLs) that impart an enhanced resistance towards conventional antibiotics [1]. They are considered a serious clinical concern due to their role in increasing the mortality and morbidity rate in infected patients [2,3]. ESBL producers are involved in urinary tract infections, intra-abdominal infections, respiratory tract infections, and bacteremia [4]. They disarm the cephalosporin class of antibiotics by acting on the β-lactam ring, and substantially affect the therapeutic regime of the infected patient [5]. In addition, ESBLs might show resistance towards other classes of antibiotics (such as tetracyclines, aminoglycosides, trimethoprim, cotrimoxazole, and quinolones) as well, which further diminishes the therapeutic options for the clinicians [6]. However, enhanced mortality in these cases was often linked with inadequate diagnosis and inappropriate antibiotic therapy [7]. In fact, close genotypic observation revealed that ESBL genes have a tendency to transpose between the different pathogens, which eventually leads to resistant infection outbreaks.

The COVID-19 pandemic has given a strong lesson to the entire world to never neglect an impending threat, whether it is small or large. Regrettably, the warning raised by eminent scientists and the WHO about antibiotic resistance has been neglected for decades. Indeed, scientists have speculated antibiotic resistance as the next major issue after the COVID pandemic, and the WHO has listed antimicrobial resistance as one of the three most significant health threats to the worldwide population in the twenty-first century. According to the WHO, most of the resistance issue arises due to the inappropriate application of antibiotics. Thus, a better therapeutic strategy with applicable antibiotic stewardship programs is urgently warranted. The failure of conventional antibiotics, either by ESBLs or by other resistance mechanisms, demands the quest for ameliorated therapeutic options. Moreover, treating infections with the lowest possible dose could be the best possible approach for effective infection control [8]. Interestingly, nanomaterials could provide a plausible approach to deliver antibiotics effectively to resistant pathogens. The nanomaterials provide a large surface area-to-volume ratio that facilitates the binding of a number of ligands (antibiotic molecules) to prepare multivalent nanoparticles against bacterial pathogens [9,10]. In addition, nanomaterials themselves exhibit antibacterial properties via hindering the formation of biofilms, triggering reactive oxygen generation, and interacting with the cell wall, DNA, and proteins of a bacterial cell. As nanomaterials do not have a defined mode of action like antibiotics, they can be very useful for tackling resistance in bacterial pathogens [11]. One such nanomaterial that has been extensively explored against resistant bacterial pathogens is gold nanoparticles (AuNPs). Due to their distinctive properties, AuNPs have been used for other biomedical applications as well, such as diagnostics, colorimetric sensing, photo-therapy, bioimaging, and gene delivery [12,13]. Furthermore, AuNP synthesis does not require complex approaches; it usually involves the addition of a metal (AuCl4 salt) precursor with an appropriate reducing and stabilizing agent [14]. AuNPs bio-synthesis could be performed by using the extracts of plants and micro-organisms [15]. However, the successful grafting of an antibiotic molecule(s) onto the surface of AuNPs involves various strategies such as physical absorption, electrostatic interactions, coupling reactions, and Au -S or -N interactions [16].

**Figure 1:**
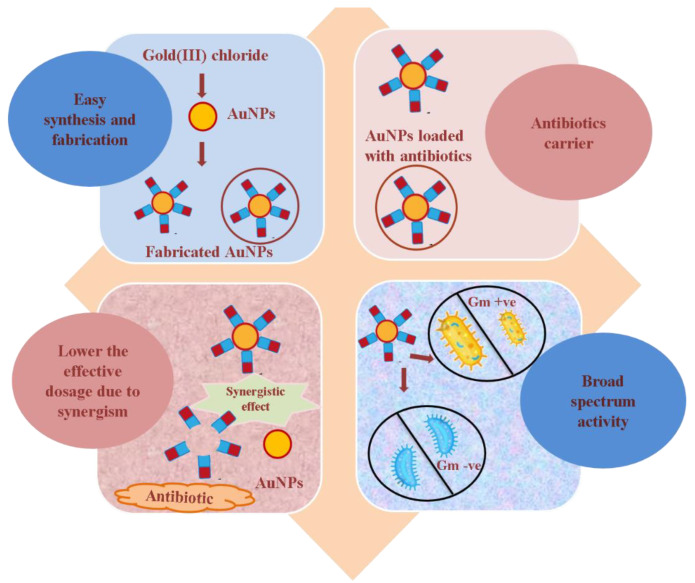
Advantages of AuNPs as a carrier of antibiotics.

AuNPs have been applied to deliver a variety of antibiotics in the past, such as ampicillin, ceftriaxone, cefotaxime, vancomycin, cefoxitin, delafloxacin, ciprofloxacin, levofloxacin, cefixime, cefotetan, colistin, amoxicillin, kanamycin, imipenem, meropenem, cefaclor, daptomycin, streptomycin, gentamycin, rifampicin, penicillin G, polymyxin B, gatifloxacin, norfloxacin, cephalexin, cefadroxil, and cefradine. These studies exhibited that the antibacterial potential of antibiotic-coated/or -conjugated AuNPs was significantly enhanced in comparison to the antibiotics alone (without AuNPs) against the tested bacterial pathogens [16,17,18,19,20,21,22,23,24,25,26,27,28,29,30,31,32,33]. Interestingly, some of these studies were conducted against highly resistant ESBL producer bacterial pathogens. In fact, AuNPs converted the unresponsive antibiotic into potent antibiotic gold nano-formulation against resistant ESBL strains. The use of AuNPs has certain advantages, such as how their synthesis and fabrication are easier, they are an excellent delivery tool for antibiotics, they lower the antibiotic dosage due to synergism, and they show broad spectrum activity (Figure 1).

However, the major bottlenecks in the clinical transformation of these findings are a lack of information on mechanistic and toxicity aspects. The present review aimed to cover all the viewpoints pertinent to the applicability of antibiotic-loaded AuNPs against ESBL- producing strains. Moreover, the review is subdivided into the following sections to provide a gist from the plethora of information without compromising the intellectual insight of the topic.

- Antibiotic-loaded AuNPs as magic bullets to overcome resistance
- Plausible antibacterial mechanism of AuNPs
- Clinical translation status and Toxicity aspects of AuNP-based drug delivery system
- Future prospects of antibiotic-loaded AuNPs
- Challenges associated with antibiotic-loaded AuNPs

## 2. Antibiotic-Loaded AuNPs as Magic Bullets to Overcome Resistance

**Figure 2:**
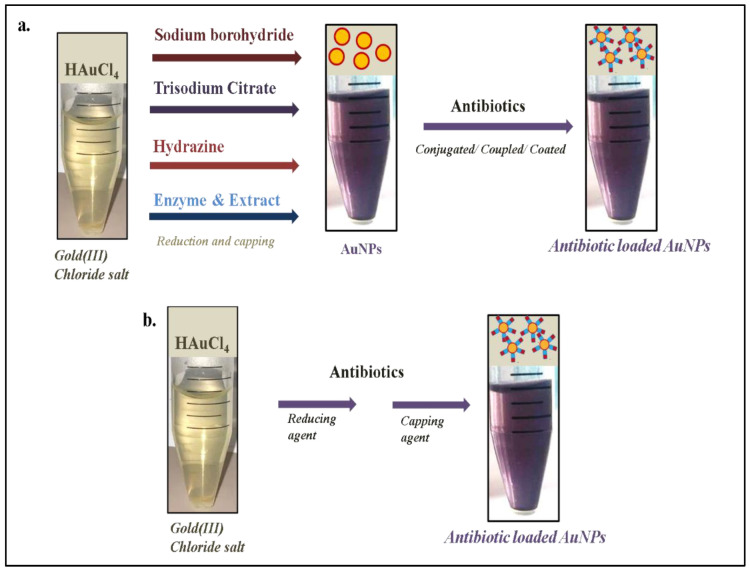
Various approaches used for the synthesis of antibiotic-loaded AuNPs. (**a**) Two or multi-step approach, (**b**) a single-step approach.

AuNPs are inorganic nanomaterials that have been widely applied in different biomedical applications due to their unique attributes [34]. In addition, AuNPs could be easily synthesized by different chemical/physical or green synthesis approaches [35]. The core of any AuNP synthesis approach includes the reduction of Au+ to Au0 by the reductant, and further stabilization with a capping agent. During chemical synthesis, chemicals are used as a reducing agent; however, in green synthesis, micro-organisms, plant extracts, and enzymes are used instead of chemicals to reduce and stabilize AuNPs [36]. Due to the localized surface plasmon resonance (or oscillations of conduction electrons after irradiation with light) of AuNPs, different colors (light pink to dark purple) can be observed that can be further correlated with shape, size, and aggregation [37,38,39,40,41,42,43,44,45]. This property of AuNPs assists the researchers in selecting the appropriate nano-formulation for further analysis. However, the grafting of an antibiotic molecule(s) makes AuNPs a useful tool to deliver antibiotics effectively to the targeted bacterial pathogens. Recently, Khandelwal et al. [16] extensively reviewed several approaches of grafting (conjugating or coating) antibiotics onto AuNPs. In short, antibiotic attachment to AuNPs involves electrostatic interaction, coupling reaction, physical absorption, and Au-S or -N interaction [16]. Khandelwal and his team identified that sodium borohydride, trisodium citrate, hydrazine, certain micro-organisms, and enzymes were often used as reducing agents for the reduction of chloroauric acid into AuNPs (Figure 2a); further, antibiotics were used to cap/or stabilize the synthesized AuNPs to obtain the desired antibiotic nano-formulation.

In the past, sodium borohydride has been used as a reducing agent for the synthesis of ampicillin, vancomycin, streptomycin, neomycin, gentamicin, and kanamycin AuNPs. However, conjugation for ampicillin was through Au-S interaction, and the authors reported that AuNPs were initially stabilized via citrate molecules, which were further replaced by a thioether moiety of ampicillin by mixing the AuNPs with ampicillin for 24 h [46]. In another study, chitosan was used as a stabilizing agent for AuNPs before preparing AuNP-stabilized vancomycin-loaded liposomes [47]. In addition, bovine serum albumin-capped AuNPs were used to prepare streptomycin, neomycin, gentamicin, and kanamycin conjugated AuNPs by physical adsorption via drop-wise addition of antibiotics in AuNPs solution [48]. On the other hand, trisodium citrate has also been explored as a reducing agent for the synthesis of ciprofloxacin, gentamicin, streptomycin, and neomycin AuNPs. Ciprofloxacin was conjugated with citrate-capped AuNPs through Au-N interaction, herein, N atom of –NH moiety of piperazine group of ciprofloxacin bound strongly onto the surface of AuNPs [49]. Similarly, gentamicin also conjugated with citrate capped-AuNPs via Au-N interactions, where the N atom of the amino group of gentamicin participated in AuNPs surface interaction [50]. Furthermore, citrate-capped AuNPs were mixed and stirred with streptomycin and neomycin to physically adsorb antibiotics on the surface of AuNPs [51]. All the above-discussed antibiotics were conjugated onto the surface of AuNPs without the use of any coupling agent, while a coupling agent such as EDC (1-ethyl-3-(-3-dimethylaminopropyl) carbodiimide) has been applied to attach gentamicin and cefotaxime to the surface of AuNPs. For gentamicin attachment through EDC, the AuNPs were initially prepared by using hydrazine as a reducing agent followed by capping with glutathione [52], whereas for cefotaxime attachment via EDC, AuNPs were synthesized using bromelain enzyme as a reducing as well as capping agent [29].

**Figure 3:**
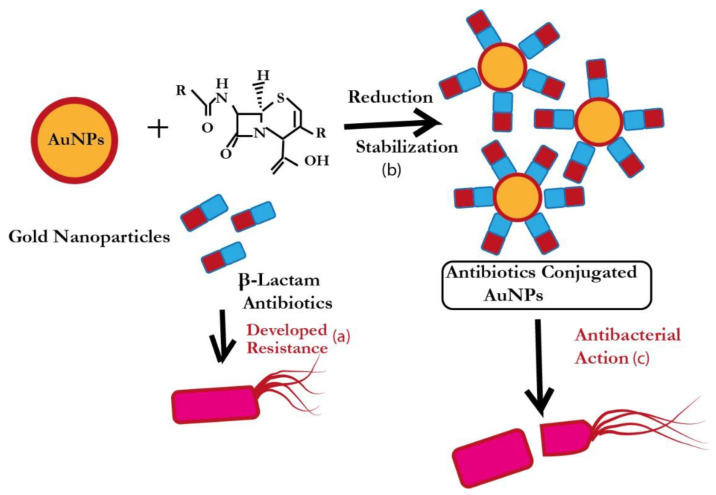
Schematic description of revival of β-lactam antibiotics by AuNPs. (**a**) Bacterial pathogens develop resistance towards β-lactam antibiotics; (**b**) AuNPs were synthesized using the same ineffective β-lactam antibiotic as reducing and stabilizing agent; (**c**) β-lactam antibiotic after loading to AuNPs become potent against the same β-lactam resistant bacterial pathogen.

Interestingly, in some reports, antibiotics themselves acted as reducing as well as capping agents (Figure 2b), such as bromelain, which eventually converted a two-step process into a facile one-step process [24,25,26,27]. An illustrative representation of the antibacterial action of revived antibiotics (after loading to AuNPs) is shown in Figure 3. This one-step strategy has actually eased the burden of the researchers working in the field of antibiotic nano-formulation and decreases the chances of doubts/errors in antibacterial results, as there is no probability of residual harmful chemicals/reducing agents (sodium borohydride, trisodium citrate, hydrazine) in the nano-formulation.

Before moving forward, it is important to discuss the influence of the sizes and shapes of AuNPs on their antibacterial activity. AuNPs could be developed in different sizes and shapes [14,53,54,55,56]. However, sizes from 5 to 70 nm are considered to possess better antibacterial properties than bigger-sized AuNPs [24,25,26,27,28,29,53]. On the other hand, most of the researchers reported the spherical shape of AuNPs as the most potent antibacterial [24,25,26,27,28,29,53], but AuNPs can be polygonal-, star-, and flower-shaped as well [54]. In contrast, one report [54] suggested that gold nanoflowers were more active and safer than spherical-shaped AuNPs.

To attain a perfect size and shape, manipulation can be performed in experimental conditions such as pH, temperature, and concentration of reducing and capping agents. Low or acidic pH is usually considered unfavorable for AuNP synthesis, as it forms unstable and aggregating bigger AuNPs. A pH between 6 and 10 is considered most appropriate for AuNP synthesis [57]. Some of the methodologies required boiling or high temperature for AuNP synthesis, but the modification of temperature conditions could influence the size of AuNPs. In some reports, it was suggested that an increase in temperature would eventually increase the size of AuNPs [14]. In fact, temperature’s influence on size depends on the type of methodology followed for the AuNP synthesis. The concentration of the reducing agent also influences the size of AuNPs; an increase in the concentration of bromelain and trypsin as reducing agents markedly increases the size of synthesized AuNPs [14,58]. Conclusively, it can be suggested that pH, if kept near to physiological pH, is better, the temperature has to be kept appropriate according to the protocol (sometimes protein is applied during synthesis and high temperature can denature it), and the amount of the reducing agent should be kept as low as possible according to the protocol.

There are numerous reports on the use of AuNPs for antibiotic delivery that are cumulated in Table 1.

However, the next section elaborates on some of the major findings on antibiotic-loaded AuNPs. The formulation of Au–silica core–shell mesoporous NPs along with silica mesoporous NPs in conjugation with amoxicillin were evaluated for the bactericidal potential against methicillin-resistant *S. aureus* (MRSA), *E. coli*, and *P. aeruginosa*. Both the stated nanocarriers played an imperative role in delivering antibiotics to the bacteria. Approximately a decline of 10 times and 20 times was reported in the effective quantity of amoxicillin against *P. aeruginosa* by using Au–silica core–shell mesoporous and silica mesoporous NPs, respectively [59]. In another study, doxycycline was conjugated with PEGylated-AuNPs, and MIC values were reduced remarkably from 32 μg/mL to 2 μg/mL against *S. aureus*, *E. faecalis*, and *E. faecium* [60].

The single-step process to synthesize AuNPs was applied in an investigation to avert the interference of reducing chemicals by using antibiotics (kanamycin, ampicillin, and streptomycin) as reducing and capping agents. The MIC concentrations of antibiotics-capped AuNPs and free antibiotics were evaluated against *S. aureus*, *E. coli*, and *M. luteus*. MIC was markedly reduced in the case of aminoglycosides after coating to AuNPs. MIC value of streptomycin was reduced from 14 to 7 μg/mL and 22 to 17 μg/mL against *E. coli* and *M. luteus*, respectively, after loading onto AuNPs. However, MIC values of kanamycin were reduced from 30 to 12 μg/mL, 32.5 to 23 μg/mL, and 9 to 5.8 μg/mL against *E. coli*, *M. luteus*, and *S. aureus*, respectively. In fact, a marked decline of 60% was observed in the case of kanamycin-loaded AuNPs in comparison to kanamycin alone [61]. Ampicillin-AuNPs in this report not showing any significance might be due to its fast precipitation from the suspension. In contrast, Chavan and his team used ampicillin (where ampicillin acted as a reducing and stabilizing agent) to produce ampicillin-capped AuNPs, and showed potent activity against an ampicillin-resistant strain of *E. coli* [18]. Additionally, ampicillin was once coupled on chitosan-capped AuNPs; it showed a 2-fold (50%) reduction in MIC values as compared to ampicillin alone against *S. aureus*, *E. coli*, and *K. mobilis* [62]. Recently, other researchers have also applied the one-step synthesis approach to produce cefaclor-, cefotaxime-, delafloxacin-, vancomycin-, and ceftriaxone-loaded AuNPs and observed marked differences in MIC against the resistant bacterial pathogens [24,25,26,63].

Fayaz and team attached vancomycin to fungal (*Trichoderma viride*) bio-synthesized AuNPs, and compared its activity with vancomycin alone against vancomycin-resistant *S. aureus*, *S. aureus*, and *E. coli* [64]. Vancomycin after attachment to AuNPs showed a reduction in MIC values from 175 to 40 μg/mL, 2 to 1.5 μg/mL, and 50 to 8 μg/mL against *E. coli*, *S. aureus*, and vancomycin-resistant *S. aureus*, respectively. In another study, vancomycin-capped AuNPs were synthesized and explored for their activity against vancomycin-resistant strains (*E. coli*, *E. faecalis*, and *E. faecium*). There is a significant decrease observed in MIC, i.e., shifted from 128 μg/mL to 2 μg/mL [65]. Like the fungal biosynthesis approach, *Rosa damascenes* petal extract was also reported for AuNPs biosynthesis, and the AuNPs were subsequently conjugated with ceftriaxone (Cef-AuNPs). The efficacies of these synthesized nanoparticles were further evaluated for their anticancer (against human breast cancer cells) and bactericidal effects (against ESBL-producing bacteria). It was found that Cef-AuNPs exhibited relatively low anticancer effects; however, conjugation of ceftriaxone on AuNPs restored the activity of ceftriaxone in otherwise resistant bacterial cells [66]. In addition, Pradeepa et al. reported the biosynthesis of AuNPs with exopolysaccharide isolated from *Lactobacillus plantarum*, and their subsequent functionalization with ceftriaxone, ciprofloxacin, cefotaxime, and levofloxacin. These AuNPs exhibited considerable bactericidal effects against multi-drug resistant *S. aureus*, *E. coli*, and *K. pneumoniae*. Among all nano-formulations, the ciprofloxacin-conjugated AuNPs were most active against *E. coli* and *K. pneumoniae*, whereas levofloxacin-conjugated AuNPs were potent against S. aureus [22]. All the antibiotics (alone) tested have MIC values of more than 10 μg/mL; however, MIC values were markedly reduced when they were grafted on AuNPs. Here, levofloxacin-loaded AuNPs were most effective against *S. aureus* (MIC: 0.562 μg/mL), ciprofloxacin-loaded AuNPs were most effective against *K. pneumoniae* (MIC: 0.281 μg/mL) and *E. coli* (MIC: 0.140 μg/mL), and ceftriaxone-loaded AuNPs were most potent against *K. pneumoniae* (MIC: 0.281 μg/mL) [22].

Most of the antibiotics discussed above belong to the β-lactams family, and can be correlated with ESBL-associated resistance, as shown in Table 1. However, some of the studies have mentioned the use of genetically identified ESBL-positive strains for antibacterial analysis. Shaikh et al. conjugated cefotaxime on bromelain enzyme-synthesized AuNPs and tested it on ESBL (CTX-M) positive strains of *E. coli* and *K. pneumoniae* [29]. Importantly, the strains tested were totally resistant to cefotaxime, and acquired sensitivity towards cefotaxime once it was loaded on AuNPs. In simple terms, AuNPs restored the potential of unresponsive cefotaxime. Here, MIC of cefotaxime after loading onto AuNPs was estimated to be 1 and 2 μg/mL against *E. coli* and *K. pneumoniae*, respectively. Recently, Alafnan et al. also applied cefoxitin-loaded AuNPs against ESBL positive cefoxitin-resistant strains of *E. coli* and *K. pneumoniae*, and observed a prominent shift of MIC from 19.5 to 1.5 μg/mL and 23 μg/mL to 2.5 μg/mL against *E. coli* and *K. pneumoniae*, respectively [26].

All these interesting reports confirmed the potency of AuNPs as a tool to combat bacterial resistance, and prompted the deciphering of the plausible mechanism of action of these antibiotic-loaded AuNPs. However, this section mainly pinpointed that antibiotics, once loaded or attached to AuNPs, become significantly active against the bacterial pathogen that was earlier resistant to the same antibiotic. This was evident from a marked decrease in MIC values against resistant pathogens. In some reports, bacterial pathogens were completely resistant at the tested concentration of antibiotic; however, once the antibiotics loaded on AuNPs at the same concentration become resuscitated, they become potent antibacterials. In addition, the methods applied for the synthesis of these nano-antibiotics were quite simple and convenient. Thus, it could be stated that the nano-conversion of antibiotics through AuNPs could revive ineffective antibiotics into potent ones.

## 3. Plausible Antibacterial Mechanism of AuNPs

The ability of AuNPs to interact with various biomolecules of the bacterial cell provides an added advantage to the antibiotic that is loaded onto them. In simple terms, AuNPs not only ease the entry of antibiotics into the bacterial pathogen, but also provide synergistic effects. There are several investigations that prove the synergism concept. In one study, *Garcinia mangostana* extract was used to bio-synthesize AuNPs, which was further conjugated with antibiotics. Biosynthesized AuNPs (without any antibiotic) did not show any significant antibacterial activity against *Pseudomonas* spp. and *Staphylococcus* spp. However, AuNPs conjugated with streptomycin and azithromycin exhibited enhanced bactericidal efficacy (33.3% and 34.8%, respectively) against *Staphylococcus* spp., as compared to free streptomycin and azithromycin. On the other hand, the conjugation of azithromycin and penicillin with AuNPs augmented their bactericidal efficacy (50% and 75%, respectively) against *Pseudomonas* spp. [67]. Furthermore, it was also reported that AuNPs in combination with antibiotics such as Clavulanate/Amoxy were effective at lower concentrations compared to the effective concentration required by Clavulanate/Amoxy or AuNPs alone [68]. AuNPs can exert their bactericidal effects by different mechanisms, and at the same time, effectively delivers a sufficient amount of antibiotic to perform their antibacterial action. An important aspect of the antibacterial action of AuNPs is that it is not properly defined. This is one sought-after advantage as bacteria usually develop resistance against a particular mechanism of action, and the development of resistance against AuNPs is quite impossible due to their undefined multivalent mode of action.

**Figure 4:**
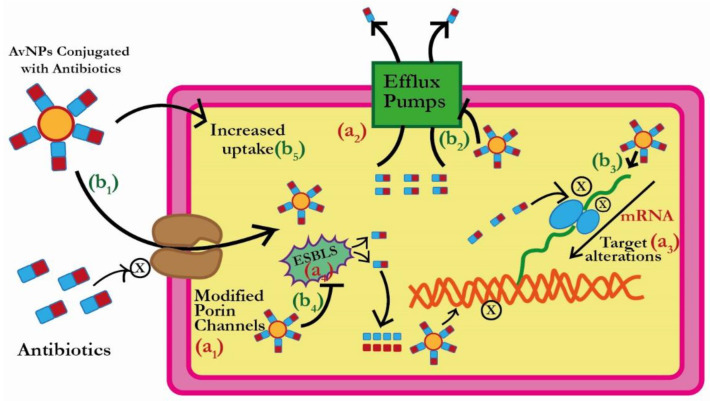
Comparison between (**a**) *bacterial resistance mechanism* and (**b**) *plausible mechanism of action of AuNPs to overcome resistance*. (**a1**) Modification of porins to hinder the entry of antibiotics, whereas (**b1**) same antibiotics, once loaded to AuNPs, gain easy entry into the bacterial pathogen; (**a2**) efflux of antibiotics from bacteria to outside of the cell, whereas (**b2**) inhibition of efflux pumps/decreasing expression of efflux pump genes by AuNPs; (**a3**) alteration of antibiotic target site, whereas, (**b3**) indirect/direct targeting of bacterial biomolecules by AuNPs; (**a4**) Antibiotic inactivating enzymes (ESBLs), whereas (**b4**) saturation of enzymes or direct damage to the enzyme structure by AuNPs. In addition, (**b5**) AuNPs can directly interact with the cell barriers, causing perforation and cell lysis.

There are four major mechanisms followed by bacterial pathogens to acquire resistance: i.e., reducing the uptake of antibiotics, effluxing antibiotics once they enter, antibiotics inactivation, and modifying antibiotic targets [69]. ESBL enzymes are mainly linked with the inactivation of antibiotics by breaking the β-lactam ring. However, reports suggested their close association with efflux pumps and reduced uptake [70,71,72,73] as well. Thus, ESBL producer bacterial pathogens can acquire resistance by three out of the four well-established mechanisms. Importantly, AuNPs can directly/or indirectly work on all these mechanisms of resistance (Figure 4). Moreover, the antibacterial action of AuNPs on each resistance mechanism has been discussed in the section below.

### 3.1. Reduction in the Antibiotic Uptake by ESBLs Producing Bacterial Pathogens

Usually, the β-lactam antibiotics enter bacterial pathogens through the porin channels [74,75,76,77]. However, bacterial pathogens modify the porin channels to reduce the uptake of β-lactam antibiotics. In a recent study, different ESBL types (TEM-1, SHV-1, CTX-M-15, OXA-48, and VIM-1) were reported in clinical pathogenic strains of *E. coli* and *K. pneumoniae*. In addition, the authors investigated the role of porins (outer membrane proteins) in these ESBL-positive strains. The porin analysis confirmed that about 95.7% and 93.3% of strains of *K. pneumoniae* and *E. coli* either modified or lost their porins. Moreover, the genetic analysis revealed the reason as mutation (frameshift), which eventually translated small-size truncated porin proteins [78]. In a similar study, the role of porins was examined for ESBL-positive strains of *K. pneumoniae* and *E. coli*, and cephalosporins (ceftazidime and cefoxitin) resistance was strongly correlated with the loss of type Omp-K35 porins [79]. In 2008, Martínez-Martínez reviewed the correlation between cell permeability and ESBLs. The author discussed the role of two porin proteins (Omp-K35 and Omp-K36) in ESBL-positive strains of *K. pneumoniae*. Interestingly, the loss of both these proteins was involved in the resistance of β-lactam antibiotics (cephalosporins, carbapenems, and ertapenem) as well as a non-β-lactam antibiotic (fluoroquinolones) [80]. Here, the role of AuNPs is to deliver the antibiotic efficiently to the target pathogens by crossing the cell barrier.

Tailored AuNPs have the ability to disrupt the cell membrane integrity and permeability. AuNPs could initially interact with the outer membrane proteins and lipopolysaccharides, and be deposited on the cell surface of the pathogens [28,81]. Further, they can slowly be translocated inside through porin channels or diffuse through the cell membrane [28,82,83]. An earlier investigation has clearly outlined the efficacy of AuNPs in instigating the disruption of bacterial cell membranes, which subsequently results in bacterial death [84,85]. Indeed, Zhao et al. investigated the potential of 4, 6-diamino-2-pyrimidinethiol (DAPT) capped AuNPs against *E. coli*. They observed that free DAPT is devoid of any antibacterial effects; however, when *E. coli* was exposed to DAPT-AuNPs, approximately 70% of the *E. coli* cells exhibited enhanced membrane permeability. Contrastingly, only 5% of the *E. coli* cells were found to be positive for such alteration of permeability in the control group. In addition, exposure to DAPT-AuNPs led to similar effects on *P. aeruginosa*. Moreover, it was observed that DAPT-AuNPs also facilitated the formation of vesicles from the outer membrane of bacterial cells. Eventually, the investigators concluded that DAPT-AuNPs exerted their bactericidal effects by altering the concentration of magnesium ions and subsequently chelating them [85,86]. Another report outlined that the divergent pattern of AuNPs aggregation was responsible for the lysis of *B. subtilis* and *E. coli* [86]. This observation was also supported by the study of Adhikari et al., where they concluded that increasing AuNPs concentration could concomitantly increase the alteration of bacterial cell permeability [87]. In one study, vancomycin alone could not pervade the outer membrane of *E. coli*; however, when added to AuNPs, it showed a promising antibacterial effect [88]. The authors suggested that AuNPs were able to perforate the cells by altering the permeability and stability of the cell membrane, thus allowing vancomycin to be efficiently delivered to the pathogen.

### 3.2. Efflux of Antibiotics by ESBLs Producing Bacterial Pathogens

Once the antibiotic enters the bacterial cells, they use efflux pumps to reduce the antibiotic load. Interestingly, efflux pumps have shown a correlation to ESBL-producing strains [70]. Maurya and his team [70], while working on ESBL-positive clinical isolates of *K. pneumoniae*, observed the overexpression of efflux pumps (belonging to the RND family) in the tested strains. They concluded that ESBLs were not the only factor against β-lactam antibiotics; efflux pumps also effectively participate in developing resistance. Another investigation also confirmed these findings by applying an efflux pump inhibitor on an ESBL-positive strain. MIC values for cefotaxime, ceftazidime, and amoxy-clavulanic acid were markedly reduced after using the efflux pump inhibitor on resistant *K. pneumoniae* [89]. It is noteworthy to mention that AuNPs can be applied against the efflux pumps as well. Recently, Dorri et al. [90] observed the effect of AuNPs on the efflux pump genes of *Pseudomonas aeruginosa*. They elucidated that AuNPs could reduce the expression of several efflux pumps on the bacterial cell surface by downregulating MexA and MexB efflux pump genes. On the other hand, Khare and his team [23] observed the significant synergistic effect of embelin-capped chitosan-AuNPs along with ciprofloxacin on the efflux pumps of *P. aeruginosa* and *E. coli*.

### 3.3. Inactivation of Antibiotics by ESBLs Producing Bacterial Pathogens

ESBL enzymes are known for the inactivation of β-lactam antibiotics. Once β-lactam antibiotics enter the ESBL-producing pathogens, ESBL enzymes disarm them by hydrolyzing the β-lactam ring. There are several reports that showed β-lactam antibiotics, once attached to AuNPs, become potent in ESBL-producing resistant strains [Table 1]. However, this change in potency against resistant strains is due to the synergistic effect of AuNPs and β-lactam antibiotics attached to them. Here, two interesting facts about the AuNP-based β-lactam antibiotic delivery system have to be noted: (1) After capping or conjugation, the active part of the antibiotic (β-lactam ring) remains intact and exposed on the AuNPs surface [16,28,91]. (2) Due to the large surface area-to-volume ratio of AuNPs, an ample amount of β-lactam antibiotic molecules can be loaded on them [92,93]. Based on these above findings, the authors proposed that the bacterial pathogens received a sufficient quantity of β-lactam antibiotic that could saturate the ESBLs, and at the same time, antibiotic molecules that remain untouched by ESBLs might start their usual action on cell wall synthesis. In addition, AuNPs have the inherent ability to alter the structure and function of enzymes via interacting with them [94,95,96,97,98]; thus, it might be speculated that AuNPs could interact with ESBLs enzyme directly and disrupt their function.

Apart from AuNPs’ interaction with bacterial enzymes/proteins, they have the ability to directly interact with bacterial DNA as well. AuNPs have been reported to enhance bacterial DNA fragmentation by 19.68% in *E. coli*, and showed an apoptotic-like cell death mechanism [99,100,101]. In addition, AuNPs could increase the expression of a caspase-like protein in *E. coli* that eventually induces ROS and SOS responses, leading to bacterial apoptosis [101]. In fact, some of the authors suggested that increased ROS generation by AuNPs might be responsible for antibacterial potential [39,102]. Contrarily, it has been suggested that instead of ROS generation, AuNPs can cause redox imbalance by reducing glutathione, and cause damage to the bacterial antioxidant system [101]. Thus, AuNPs have the ability to attack the pathogen by alternate pathways, even if the bacterial pathogen has modified the antibiotic target site.

This section deals with the mechanistic aspect of the antibacterial action of antibiotic-loaded AuNPs. The reports revealed that AuNPs not only deliver the antibiotic successfully to the resistant pathogen, but work in synergy with the antibiotic to increase the antibacterial effect. In addition, after the loading of antibiotics onto the AuNPs, the resistance mechanism of the pathogens could be overcome in various ways. In fact, AuNPs could work smartly on each antibiotic resistance mechanism of bacterial pathogens to provide the maximum antibacterial effect. Bacterial pathogens have the ability to develop resistance against the defined mechanism of action of any antimicrobial agent with time; thus, the multi-targeting ability of AuNPs or undefined mechanism of action could be considered a blessing in disguise. The present review tried to propose the best plausible mechanism of action of AuNPs (or antibiotic-loaded AuNPs) based on previous investigations. However, the work on the exact mechanism of action of antibiotic-loaded AuNPs is still obscure and needs to be deciphered.

## 4. Clinical Translation Status and Toxicity Aspects of AuNP-Based Drug Delivery System

### 4.1. Clinical Translation Status

Before discussing the clinical translation status, glimpses of some recent global market trends about AuNPs need to be shared. The global market of AuNPs has reached about USD 4.4 billion in the year 2021, and is further expected to reach around USD 8 billion by the year 2027 [103,104]. Several companies such as Sigma-Aldrich, Goldsol, Cytodiagnostics, NanoHybrids, ParticleWorks, BBI Solutions, and Metalor Technologies are applying AuNPs for various purposes [105]. These market trends directly suggest that AuNPs have enormous potential if utilized appropriately. AuNP-based Verigene^®^ system has already obtained approval and is used for genotyping different genes in DNA samples [106]. There are so many clinical trials going on to establish an AuNP-based therapeutic system. Some are in Phase I, such as pro-insulin functionalized on AuNPs (C19-A3 AuNP), T-cell priming cocktails of dengue virus peptides functionalized on AuNPs (naNO-DENGUE), TNF functionalized on PEGylated AuNPs (CYT-6091), and T-cell priming cocktails of coronavirus peptides functionalized on AuNPs (naNO-COVID); however, CNM-Au8 (AuNP in drinkable bicarbonate solution) is in Phase II of clinical trials [NCT04935801]. These AuNPs are applied in different diseases, such as C19-A3 AuNP in autoimmune diabetes, naNO-DENGUE in dengue fever, CYT-6091 in tumors, naNO-COVID in COVID-19, and CNM-Au8 in neurodegenerative diseases [NCT00356980]. There are some other examples as well, such as AuroShell (for different cancer types), NU-0129 (for glioblastoma), and Nanoshell (for atherosclerotic plaques), that are undergoing different stages of clinical trials [107]. Although antibiotic-conjugated AuNPs have not undergone clinical trials, there is a strong probability that they will soon be applied in various antibacterial preparations. However, the lack of investigations on the toxicity aspects of AuNPs and doubt about their fate in the human body are considered major obstacles to the successful conversion of lab-bench findings into bedside medications. The next section covers the toxicity aspects of AuNPs.

### 4.2. Toxicity Aspects

The scientific literature surveyed in the present review indicated that in the majority of investigations, AuNPs were found to be relatively less toxic chemically. Nevertheless, certain reports have also outlined the potential toxicity of these AuNPs, which is critically governed by the charge, shape, surface chemistry, and size of the synthesized AuNPs. A previously published report elucidated that AuNPs conjugated with ligands, namely Ph_2_PC_6_H_4_SO_3_Na and P(C_6_H_4_SO_3_Na)_3_, showed cytotoxic effects against different cells. It was further shown that smaller AuNPs exhibited approximately 60-fold enhanced cytotoxic effects compared to 15 nm size AuNPs. During an extended study, the same research group demonstrated that AuNPs capped with triphenylphosphine mono-sulfonate (size of 1.4 nm) induced the production of reactive oxygen species (ROS), which subsequently augmented inflammatory mediators resulting in the onset of mitochondria-mediated apoptotic cell death in human cervical carcinoma HeLa cells [108]. Indeed, a report from a different lab also showed that AuNPs having a size of 1.4 nm exhibited high binding affinities towards the DNA of normal as well as cancer cells [109].

Studies by Mironava et al. made it evident that AuNPs having a radius of 13 nm–45 nm fail to enter either the mitochondria or nucleus of human-derived dermal fibroblast cells. Furthermore, their report also indicated that AuNPs within the stated range are localized within the cytoplasmic vacuoles, and AuNPs of around 45 nm instigated increased cellular damage owing to their different uptake and release mechanisms in the cytoplasm [110]. Contrastingly, the shape of AuNPs also plays an important role in regulating drug delivery. It has been demonstrated that spherical AuNPs are more efficiently absorbed than rod-shaped AuNPs [111]. Another important aspect regulating nanoparticle-mediated drug delivery is the charge of nanoparticles. Goodman et al. explicitly showed that nanoparticles bearing a positive charge exert enhanced cytotoxic effects in comparison with negatively charged nanoparticles [112]. In addition, some reports have indicated that the surface chemistry of AuNPs is a more effective mediator of AuNPs toxicity than the charge of AuNPs (alone). This notion is also supported by a study where it was observed that positively charged poly(diallyldimethyl ammonium chloride)-coated AuNPs were more bio-compatible with the cell membrane in comparison to positively charged cetyl trimethylammonium bromide coated AuNPs [113].

Interestingly, some literature has outlined the importance of AuNPs’ size and charge in regulating their biodistribution and absorption in different animal models. Several studies concluded that small AuNPs with a negative charge exhibit the highest absorption rate, and thus have distribution in an array of organs within the animals [112,113,114,115]. In addition, it has become evident that the shape and capping of AuNPs have also affected their bio-distribution in animal models [115,116]. In contrast, studies have reported the accumulation of AuNPs in the liver and spleen irrespective of their surface chemistry and charge [116,117]. In fact, kidney homeostasis was also affected by exposure to AuNPs. AuNPs also induce size-dependent damaging effects in renal cells where smaller particles have substantial damage in comparison to large particles [116,117,118]. Moreover, it is now well-established that the route of administration of AuNPs could influence the cytotoxic effects in model animals. It was previously reported that the toxic effects of AuNPs were aggravated after oral and intraperitoneal administration in comparison to administration via the tail vein [119].

It is to be noted that AuNPs genotoxicity has been evaluated in Zebra fish models, and administration of positively charged N,N,N-trimethylammoniumethane thiol stabilized AuNPs instigated disruption in development and pigmentation of the eyes that was concomitantly followed by impaired behavioral attributes with distinctive neuronal damage [120]. Moreover, a previous report has substantiated that AuNPs having a size of 15 nm were competent in inducing phenotypic mutations within generations of *Drosophila melanogaster*, thereby indicating that mutagenic effects of AuNPs can be further transmitted to progenies [121,122]. It is thus evident that there are a large number of contradicting reports available in the scientific database regarding the toxicity of AuNPs; therefore, it is difficult to generalize their important toxicity issues. The investigations reported in the present review indicated some evident issues regarding AuNPs toxicity; nevertheless, it also showed that the toxicity of AuNPs depends collectively on the preparatory methods and physiochemical attributes of AuNPs. Moreover, before concluding any holistic inferences, more elaborative investigations are further warranted for an in-depth understanding of mechanisms regulating the changes in physiochemical aspects of AuNPs within a living system.

This section summarizes the clinical translation status and toxicity aspects of AuNPs. Companies are investing in AuNPs for different applications, and clinical trials are going- on in different phases. Most of the clinical trials have been conducted on fabricated AuNPs against non-infectious diseases, and some are conducted for viral infections. To date, antibiotic-loaded AuNPs have not reached any of the phases of clinical trials. Nevertheless, the results of fabricated AuNPs clinical trials have provided some strong optimism for AuNP-based therapy. Toxicity, biodistribution, and targeting are major obstacles in any drug development process. It is evident from the reports that AuNPs toxicity and distribution majorly depend on the fabrication or modification of the surface. However, antibiotic-loaded AuNPs have not been thoroughly studied for toxicity and biodistribution. Thus, the toxicity of antibiotic-loaded AuNPs could be considered one of the translational gaps in AuNP-based nano-antibiotic development.

## 5. Future Prospects of Antibiotic-Loaded or -Conjugated Gold Nanoparticles

Till now, this review has substantially compiled and discussed the published research and progress pertinent to antibiotic-loaded AuNPs. However, the question still remains the same: ‘Could AuNPs be clinically applied to tackle the superbugs threatening our life?’. The answer to this question will not just be confined to simply saying yes or no. It has been clearly discussed in the above sections that AuNPs are versatile in their ways of interactions with bacteria and their cellular components, and further, their control over their resistance mechanisms. Interestingly, there are various reports suggesting AuNPs’ potential as an efficient tool to deliver antibiotics to the pathogen and significantly reduce its effective MIC concentration. However, the mode of action of antibiotic-loaded AuNPs still needs to be fully deciphered and validated. In addition, a substantial focus has to be shifted to deciphering the safety of AuNP-based antibiotic delivery systems. In fact, long-term in vitro and in vivo biosafety experiments have to be designed to translate and guide the successful transformation of research findings into clinical applications. Standardization of experimentations is another area that requires significant improvement in order to perform comparative studies. Nevertheless, it is also a fact that AuNPs are presenting new hope in the current scenario, where clinicians have become helpless against resistant bacterial infections due to the lack of availability of effective therapeutic options. It is strongly anticipated that AuNP-based therapeutic options against antibiotic-resistant pathogens have the capability to overcome resistance from society if utilized in an effective manner.

## 6. Challenges Associated with Antibiotic-Loaded or -Conjugated Gold Nanoparticles

It is quite clear from the earlier sections that AuNPs could be easily fabricated and loaded with the desired antibiotic(s) by various approaches. In addition, their complete characterization before and after the loading of antibiotics has become convenient nowadays. Moreover, there is a plethora of research that confirmed antibiotic-loaded AuNPs as smart and effective antibacterial agents against resistant pathogens. However, there are still certain challenges that have to be overcome before considering them as the best arsenal against resistant pathogens. The major challenges are as follows: (1) the safety of antibiotic-loaded AuNPs; (2) large-scale production and processing; and (3) cost-effectiveness over antibiotic treatment.

There are several clinical trials going on to evaluate the potential of AuNP-based therapy, but none of them are on antibiotic-loaded AuNPs. The safety profile of antibiotic-loaded AuNPs is not fully investigated, and animal model evaluation studies are still undergoing. More biodistribution and pharmacokinetics studies on these antibiotic-loaded AuNPs have to be designed, as it has been observed that AuNPs have a tendency to aggregate at the site of inoculation. In addition, mistargeting is one of the aspects linked with nanoparticles; hence, this point also needs to be considered while designing antibiotic-loaded AuNPs.

The scenario of large-scale production of nanomedicine is quite different from the laboratory level. Large-scale production requires cost-effective approaches, large equipment, skilled technicians, and a quality control/assurance unit. Although many big industries have started investing in AuNPs for various applications in the last decade, pharma giants also need to step up and show interest in the development of nano-antibiotics from antibiotic-loaded AuNPs. In addition, different approaches for the conversion of these AuNPs from lab scale to industrial scale in the best possible way have to be developed.

The most important question is the cost effectiveness of AuNP-based antibiotics compared to available antibiotic options. AuNP development is itself a costly affair; hence, ways need to be developed to reduce the cost of AuNP-based nano-antibiotics formulation.

Earlier, Zhang et al. [123] nicely reviewed the antimicrobial activity of AuNPs, and covered some aspects of the antimicrobial activity of AuNPs in combination with antibiotics. However, the present review provided detailed information specifically on antibiotic-loaded or conjugated-AuNPs. The review covered the synthesis aspect, provided an opinion on the best possible way of synthesis, tried to cover almost all antibiotic-loaded AuNPs to date, designed the plausible mechanism of action based on the literature, experiences, and understanding, suggested the mechanism to overcome resistance in pathogens, and discussed the bottlenecks for clinical translation and future prospects. All the information pertinent to the above-mentioned topics from July 2001 to November 2022 has been collected, reviewed, interpreted, and summarized on one platform exclusively for antibiotic-loaded AuNPs. In fact, these aspects make the review quite different from the earlier reviews conducted on AuNPs.

## 7. Conclusions

The advent of nanotechnology has seen a surge in its advancement and usage to cater to the growing need for clinical interventions, primarily in cases of infectious diseases due to the increasing global prevalence of antibiotic resistance. Undoubtedly, conjugation/coating of several antibiotics onto AuNPs has demonstrated elevated antibacterial efficacy against resistant bacterial pathogens. Indeed, simple alteration in the attributes of AuNPs has allowed the investigators to develop novel antibacterial formulations that could plausibly be applied in clinical settings. The mechanistic aspect of the antibacterial action of AuNPs revealed that AuNPs could help to mitigate each and every perspective of antibacterial resistance. Importantly, preclinical findings on AuNPs are gradually moving towards clinical evaluations, and R&D sections of big industries are showing keen interest in these updates. Still, mechanistic details of its action, its fate inside the human body, and biosafety are the segments that need special attention. However, AuNPs are one of the most promising hopes against antibiotic resistance that require proper exploration to be translated from bench to bedside.

## Figures and Tables

**Table 1:** Antibiotic-loaded AuNPs against different bacterial pathogens.

Nanomaterial	Size and Shape	Targeted Bacteria	Efficacy	Remarks	Ref.
**Vancomycin-AuNPs**	Polygonal	*Vancomycin-resistant* *Enterococci*	MIC_50_ of Vancomycin-AuNPs was 2 µg/mL, which was significantly lower than free vancomycin	AuNPs developed have the ability of photothermal killing by irradiation	[17]
**Ampicillin-** **AuNPs**	25–50 nm and spherical	*S. aureus*, *E. coli*, *B. subtilis*, *Flavobacteria* *devorans*	4- and 16-fold increase in activity of ampicillin- AuNPs against amp-resistant and amp-sensitive strains, respectively	Ampicillin-mediated AuNP synthesis, where β−lactam ring remains intact for its action after attachment	[18]
**Colistin-** **AuNPs**	5 nm and spherical	*E. coli*	6-times reduction in MIC concentration compared to free colistin	Anionic AuNPs were used to deliver colistin that showed activity at very low dose compared to colistin alone	[20]
**Azithromycin/Streptomycin** **with AuNPs**	20–40 nm and spherical	*Clinical Staphylococcus* spp.	Significant improvement in antibacterial activity in comparison to free antibiotics	Synergistic effect was observed after combining AuNPs with antibiotics	[21]
**Ciprofloxacin- AuNPs**	10–20 nm and spherical	MDR *K. pneumoniae* MDR *E. coli*	Synergistic antibacterial effect	Bacterial efflux pump is targeted along with usual antibacterial action	[22,23]
**Cefotaxime-** **AuNPs**	65 nm and spherical	*E. coli*, *K. oxytoca*, *S. aureus*, *P. aeruginosa*	Marked reduction in MIC concentration was observed against all the tested strains in comparison to free cefotaxime	Cefotaxime-mediated AuNP synthesis, where more than 80% of cefotaxime was loaded and showed potent activity against both gm + ve and gm -ve bacteria	[24]
**Ceftriaxone-** **AuNPs**	21 nm and spherical	*E. coli*, *S. aureus*, *S. abony*, *K. pneumoniae*	Significantly (2-fold) better potential against the tested strains in comparison to free ceftriaxone	Ceftriaxone-mediated AuNP synthesis, where 79% of ceftriaxone was loaded and showed potent activity against both gm +ve and gm -ve bacteria	[25]
**Cefoxitin-** **AuNPs**	2–12 nm and spherical	ESBL +ve *E. coli*, *K. pneumoniae*	Marked potency against cefoxitin-resistant strains of *E. coli* and *K. pneumoniae*	Cefoxitin-mediated AuNP synthesis, where 70% of cefoxitin was loaded and showed potent activity against uropathogenic resistant gram -ve strains	[26]
**Delafloxacin -AuNPs**	16 nm and spherical	*E. coli*, *P. aeruginosa S. aureus* *B. subtilis*	Potent antibacterial activity against all the tested gram +ve and -ve strains in comparison to free delafloxacin	Delafloxacin-mediated AuNP synthesis, where around 90% of delafloxacin was loaded and showed more potent activity against gram -ve strains as compared to gram +ve strains	[27]
**Cefaclor-** **AuNPs**	22 nm and spherical	*S. aureus*, *E. coli*	Marked increase in potency against both the tested strains	Cefaclor-mediated AuNP synthesis, where AuNPs form pores in the cell wall and ample cefaclor was available for its antibacterial action	[28]
**Cefotaxime-** **AuNPs**	17.55 nm And spherical	CTX-M-15 positive *E. coli*, *K. pneumoniae*	Increased potency against CTX-M-15 positive resistant bacterial strains	Cefotaxime was conjugated on bromelain synthesized AuNPs with the help of coupling agent EDC and showed potent activity against resistant gram -ve strains	[29]
**Imipenem/Meropenem-** **AuNPs**	35–200 nm	*K.pneumoniae*, *P.mirabilis*, *A. baumanii*	Marked augmentation in antibacterial activity against all the tested strains	Carbapenem antibiotics were loaded on citrate stabilized AuNPs, and reduced the MIC of Imipenem by 4-fold and meropenem by 3-fold	[30]
**Amoxicillin-** **AuNPs**	33.9 nm	*Methicillin-resistant* *S. aureus*	Enhanced potency against MRSA, and less cytototoxicy in in vivo study	Amoxicillin was loaded on herbal synthesized AuNPs and showed potency to overcome β-lactamase-mediated resistance in MRSA	[31]
**Vancomycin-** **AuNPs** **and Vancomycin** **AgNPs**	11 nm	*Methicillin-resistant* *S. aureus*	2.4- to 4.8-fold increase in antibacterial activity of AgNPs than AuNPs against MRSA	Vancomycin AgNPs were more effective than vancomycin AuNPs against MRSA	[32]
**Daptomycin-AuNPs**	80 nm	*E. coli*, *S. aureus*	Antibacterial inhibition rates were 64% and 52% for *S. aureus* and *E. coli*, respectively	Near-infra-red radiation caused significant photothermal inhibition of bacterial growth	[33]

## Data Availability

Not applicable.
